# Lemierre’s Syndrome Due to Streptococcus anginosus: A Case Report and Review of the Literature

**DOI:** 10.7759/cureus.44311

**Published:** 2023-08-29

**Authors:** Nihar Jena, Prashanth Reddy Yella, Deepak Chandramohan

**Affiliations:** 1 Cardiovascular Medicine, Saint Joseph Mercy Oakland, Pontiac, USA; 2 Internal Medicine/Hospital Medicine, Yuma Regional Medical Center, Yuma, USA; 3 Nephrology, University of Alabama, Birmingham, USA

**Keywords:** anticoagulation, antibiotics, lemierre’s syndrome, septic thrombophlebitis, streptococcus anginosus

## Abstract

Lemierre’s syndrome, also known as anaerobic post-anginal septicemia, necrobacillosis, and the “forgotten disease,” is a rare manifestation. It is often presented with sepsis, sore throat, fever, neck pain, internal jugular vein thrombophlebitis/thrombosis, and septic emboli. The bacteria that are usually associated with the disease are *Fusobacterium* species, but it is also associated with* Staphylococcus*, *Streptococcus*, and other bacterial species. The diagnosis of Lemierre’s syndrome is made based on evidence of septic thrombophlebitis, preceding oropharyngeal infection, and positive culture. Treatment usually consists of antibiotics directed toward the causative organism. The use of anticoagulation, although controversial, is shown to be beneficial by several studies. We describe a middle-aged patient who presented with a sore throat, neck pain, and dysphagia. Imaging of the neck and chest revealed right jugular thrombosis along with septic emboli in the lungs. The culture of the blood and pus drained from the peritonsillar abscess grew *Streptococcus anginosus*. In this study, we have illustrated the effective management of Lemierre’s syndrome with antibiotics, anticoagulants, and needle aspiration of abscess.

## Introduction

Lemierre’s syndrome is characterized by complicated bacterial pharyngitis and tonsillitis that causes septic thrombophlebitis in the internal jugular veins. It is a rare disease with an incidence of 3.6 cases per million per year. Historically, it was first studied by André Lemierre, who reported a series of 20 cases with the syndrome that had a 90% mortality rate. The incidence of the disease has decreased in recent decades due to the widespread use of antibiotics, so many referred to it as a “forgotten disease” [1]. However, in recent years, cases have reemerged, and a meta-analysis reported that there has been an increase, possibly attributable to emerging resistance to antibiotics and reluctance of antibiotic use in pharyngitis. The predominant pathogen implicated in the disease is *Fusobacterium necrophorum*, an anaerobic gram-negative bacillus. Infections from other organisms, such as *Staphylococcus*, *Streptococcus*, and other bacterial species, have also been reported. The use of anticoagulation to treat thrombus formation is a topic of debate. The paucity of data on anticoagulation and the lack of guidelines complicate the decision to choose anticoagulant therapy [1,2]. This study reports a case of Lemierre’s syndrome in a middle-aged male with pharyngitis and dysphagia managed with needle aspiration of abscess, antibiotics, and anticoagulation. A *Streptococcus anginosus* association further enhances the unusual aspect of this case.

## Case presentation

A 56-year-old African American male with a past medical history of diabetes mellitus presented to the hospital with a chief complaint of sore throat, dysphagia, and right-sided facial and neck pain. His symptoms started three days prior to hospital admission as a sore throat. He described the pain as dull aching type, mild, 1-2 out of 10 in intensity, on the right side of his oropharynx, radiating to the right upper neck, and mild subjective fever. His pain progressively worsened, associated with dysphagia, especially with solid food, severe odynophagia, decreased oral intake, subjective fevers, chills, and mild shortness of breath. He denied recent trauma or any procedure to the neck. There was no history of smoking, drinking, or recreational drug use. At presentation, he was febrile, tachycardic, and tachypneic. His vital signs are noted in Table 1.

**Table 1:** Vital parameters at presentation

Vital parameters	Patient’s findings	Reference range
Blood pressure	125/62 mmHg	90-120/60-80 mmHg
Heart rate	134 bpm	60-100 bpm
Respiratory rate	43 breaths/minute	12-18 breaths/minute
Oxygen saturation	93% on room air	95%-100% on room air
Temperature	103.3°F	97°F-99°F

**Figure 1:**
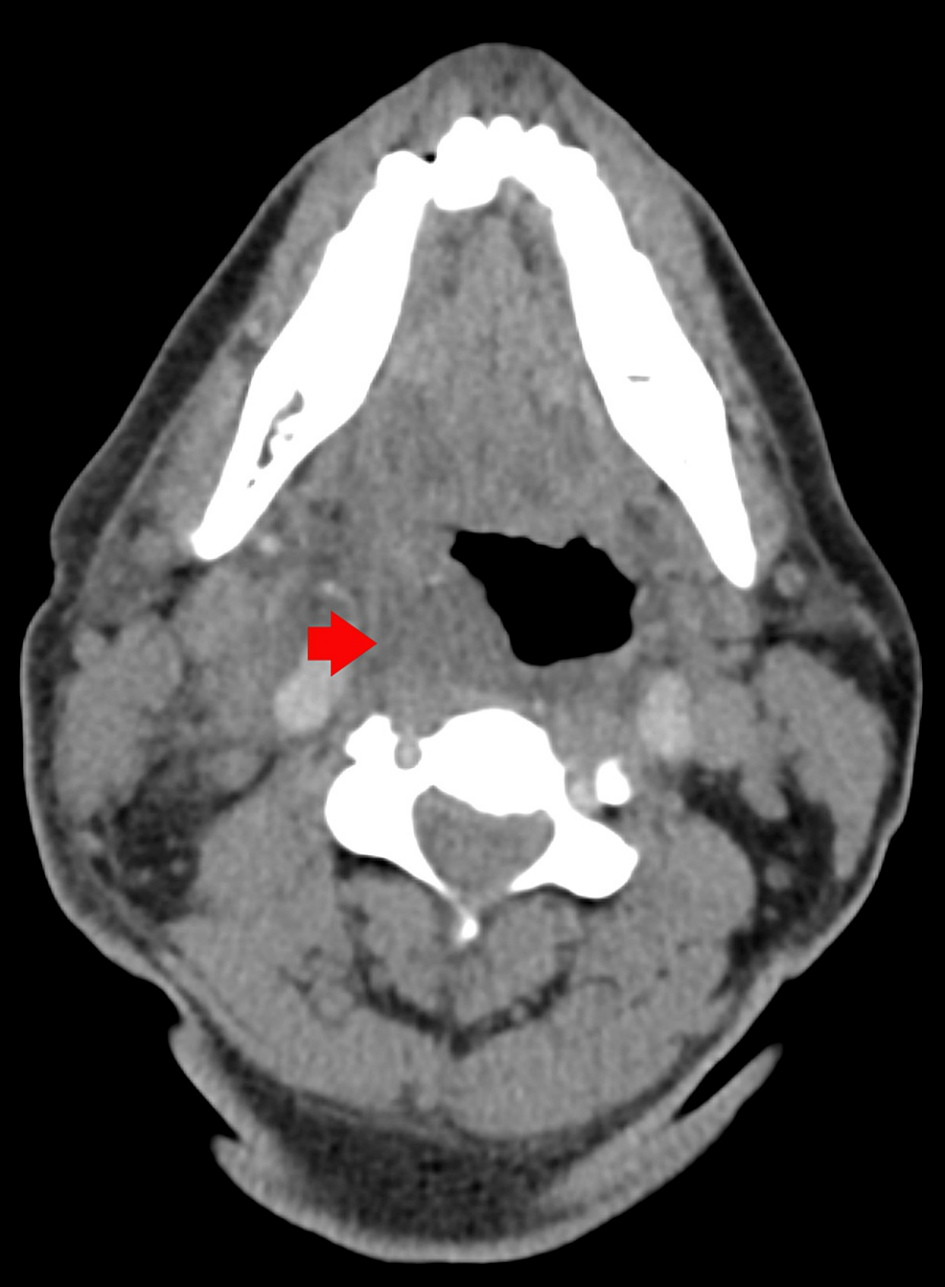
Computed tomography of the neck and soft tissue showing peritonsillar edema and possible abscess formation (red arrow)

**Figure 2:**
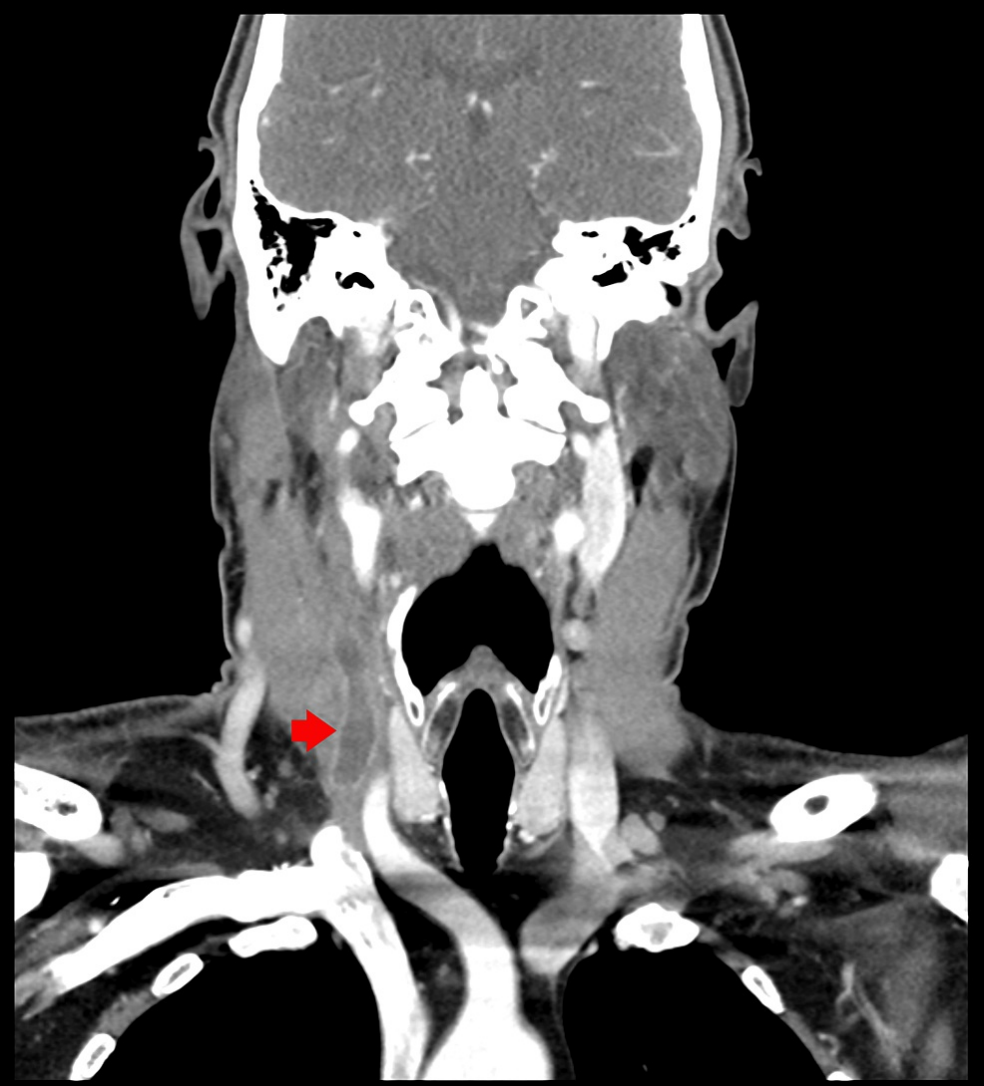
Computed tomography scan of the neck and soft tissue with contrast showing right-side internal jugular vein thrombosis (red arrow)

His general physical examination was notable for an ill-looking patient in mild distress, with mild swelling on the right side of his neck, without pallor or cyanosis. He was tachypneic, hypoxic, tachycardia, and febrile. His ear, nose, and throat examination showed oropharyngeal erythema with congestion on the right side and a slight deviation of the uvula toward the left. The larynx revealed good mobility bilaterally. The patient had edema from his nasopharynx to his hypopharynx on the right. The airway examination revealed no drainage or membranes, and the airway was not obstructed. The rest of the physical examination was unremarkable. The patient’s initial laboratory work was notable for leukocytosis with a left shift, mildly elevated liver enzymes, acute kidney injury, and elevated lactate levels (Table 2). Due to the above presentation with the laboratory works, the patient was admitted for a working diagnosis of severe sepsis with a source of infection as a possible peritonsillar abscess. Therefore, blood cultures were drawn, and a computed tomography (CT) scan of the neck with contrast showed the presence of thrombus/thrombophlebitis in the right internal jugular vein and soft tissue prominence in the right peritonsillar region (Figure 1, Figure 2).

**Table 2:** Laboratory results WBC: white blood count, CO2: carbon dioxide, ALP: alkaline phosphatase, ALT: alanine transaminase, AST: aspartate aminotransferase, BUN: blood urea nitrogen

Laboratory results	Patient’s results	Reference range
WBC	11.7 K/mcL	3.7-11 K/mcL
Hemoglobin	11.8 gm/dL	12-16 gm/dL
Platelet counts	149 K/mcL	140-440 K/mcL
Sodium	135 mmol/L	135-144 mmol/L
Potassium	3.5 mmol/L	3.5-3 mmol/L
Chloride	103 mmol/L	98-107 mmol/L
CO2	18 mmol/L	21-31 mmol/L
Calcium	8.4 mg/dL	8.6-10.3 mg/dL
ALP	209 U/L	27-120 U/L
ALT	81 U/L	7-52 U/L
AST	91 U/L	13-39 U/L
Total bilirubin	1.8 mg/dL	0.3-1 mg/dL
BUN	31 mg/dL	7-25 mg/dL
Creatinine	1.86 mg/dL	0.60-1.20 mg/dL
Lactate	3.2 mmol/L	0.5-2.2 mmol/L

**Figure 3:**
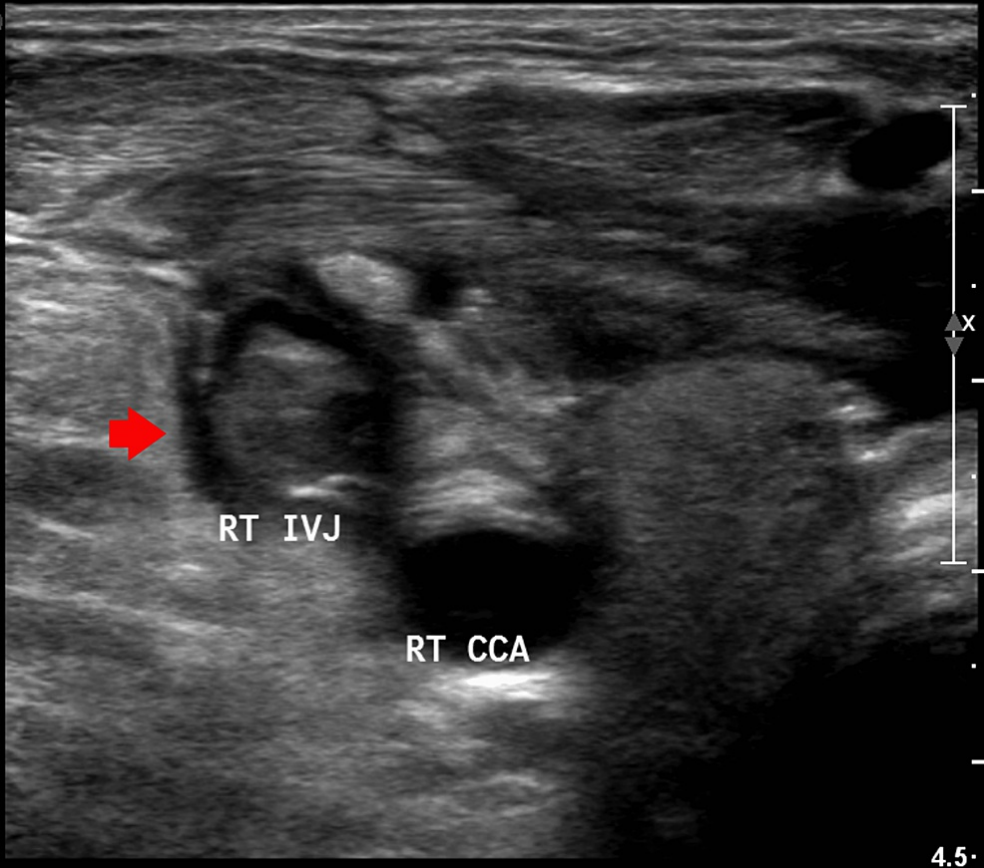
Doppler ultrasonogram of the right internal jugular vein showing echogenicity (red arrow) indicating the presence of thrombus RT IVJ: right internal jugular vein, RT CCA: right common carotid artery

A Doppler ultrasound of the neck was performed, displaying the non-compressibility of the right internal jugular vein with echogenicity confirming the presence of thrombus (Figure 3).

**Figure 4:**
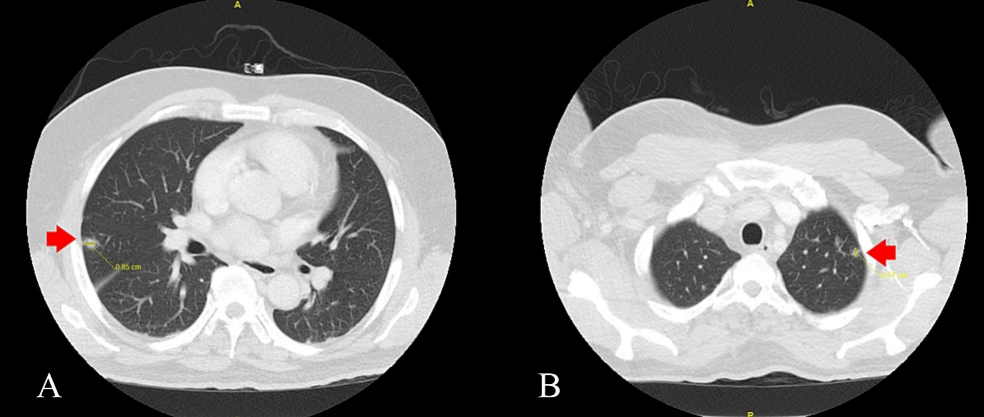
A and B: Computed tomography scan of the chest showing septic emboli in the right and left lung (red arrows)

CT scan of the chest was obtained, showing septic emboli measuring 0.85 cm in the right lung and 0.57 cm in the left lung (Figure 4A, Figure 4B).

In this case scenario, differential diagnosis is limited to infectious etiologies. The most common infectious etiology is viral. Most cases are not severe enough to present to the hospital, and an abscess negates this differential. Sometimes, pulmonary malignancy can present with low-grade fever and venous thrombosis. Imaging the chest did not suggest any malignancy in the patient, and abscess formation is not typical in lung malignancy. The patient was started on ampicillin/sulbactam and vancomycin as broad-spectrum antibiotics, which were later de-escalated to ceftriaxone and metronidazole based on the culture result. Subsequently, an ear, nose, and throat (ENT) specialist was consulted after the CT scan of the neck showed right internal jugular vein thrombophlebitis. Needle aspiration of the peritonsillar abscess was performed with drainage of purulent material, which was sent for culture. He also received 10 mg of dexamethasone to decrease the swelling and prevent airway compromise. Lemierre’s syndrome was diagnosed after the Doppler of the neck showed the right internal jugular vein thrombus, and the patient was started on heparin infusion to prevent further propagation of the thrombus and complications. The blood culture result returned positive for *Streptococcus anginosus*; the culture of the drained purulent fluid grew the same organism beside anaerobic gram-negative bacilli, which were later identified as contaminants.

After the procedure, the patient had no further episodes of fever, and his white blood cell count downtrended. After the needle aspiration, the patient was monitored for one night in the critical care unit and subsequently triaged to the general medical floor. He was switched from unfractionated heparin to apixaban. The patient made a full recovery within five days of the hospital admission. He was advised to continue ceftriaxone and metronidazole for three more weeks and apixaban for eight weeks.

He was followed up in the outpatient clinic one week and four weeks after hospital discharge. CT scan of the neck and soft tissue was performed in four-week intervals, which showed improved right peritonsillar swelling and resolving thrombus. Then, a phone interview was performed within six months duration. He denied any further symptoms.

## Discussion

Recent advancements in antibiotics have reduced the current incidence of Lemierre’s syndrome. As antibiotic stewardship has gained traction, few cases of missed bacterial pharyngitis have led to presentations of this rare condition. Although the occurrence is rare, its presentation is fatal. It is a severe inflammatory condition characterized by sore throat, thrombophlebitis of the internal jugular veins, and isolation of the anaerobic pathogens. *Fusobacterium necrophorum* is the usual causative organism, an anaerobic gram-negative bacterium found commonly in the oropharynx. Many authors do not consider the isolation of *Fusobacterium* an essential criterion to diagnose Lemierre’s disease as it is difficult to culture the organism, and most patients get antibiotics before collecting specimens for culture. In their review article, Karkos et al. [3] reported that 90% of cases are due to *Fusobacterium* species, and 10% are due to *Streptococcus* and other gram-negative anaerobes. Similarly, Chirinos et al. [4] depicted an occurrence of 81.7% of cases by *Fusobacterium necrophorum*, 5.5% with other organisms, and 12.8 % with negative cultures. The other less common bacterial species include *Streptococcus*, *Staphylococcus*, and *Klebsiella*. *Streptococcus anginosus* as the causative agent has been reported sparsely. Several cases have been reported as causative organisms, such as *Streptococcus anginosus*, and one such case was published by Osman et al. [5]; the case described a 59-year-old patient with dysphagia and right-side neck swelling successfully treated with surgical drainage of abscess and antibiotics. The most common age group involved is young adults aged 16-24 years [6]. Patients present with pharyngitis in 82.5% of cases. Other clinical features such as otalgia, fever, swollen neck, and cervical lymphadenopathy are not uncommon [4]. The first phase of the disease is typically pharyngeal inflammation, leading to odynophagia. The second phase involves infection invasion to the lateral pharyngeal wall and subsequent thrombophlebitis of the internal jugular vein. The metastatic infection used to cause cavitation pneumonia and arthritis, which is rare nowadays.

Diagnosing this forgotten entity requires high clinical suspicion and radiographic evidence. Ultrasound of the neck vein is a simple and inexpensive way to identify jugular vein thrombosis, but it carries a lower sensitivity. Magnetic resonance imaging is superior to visualizing the anatomical structures and the presence of thrombus but is cost-prohibitive. Computed tomography scan has higher sensitivity than ultrasonogram, is readily available, and is usually the preferred method, but it is associated with radiation exposure [7]. Both imaging modalities have the advantage of showing the oropharyngeal abscess, which is crucial to diagnosing Lemierre’s disease. It is essential to consider clinical and serological markers before proceeding to the image, which can help stabilize vitals and initiate early antibiotics.

The widespread use of antibiotics has changed the original description of the disease’s progression. Prompt identification and treatment with appropriate antibiotics are essential to break the sequel of the fatal disease. In most cases, penicillin, carbapenems, or piperacillin/tazobactam combined with metronidazole are sufficient [8]. Antibiotics need to be tailored based on culture findings. The duration of antibiotics varies between 10 days to eight weeks, but the mean duration is four weeks. In this case, the patient was started with broad-spectrum antibiotics with ampicillin/sulbactam and vancomycin. After the culture returned positive for *Streptococcus anginosus* and anaerobic bacteria, he was switched to ceftriaxone and metronidazole. We decided to treat for a total duration of four weeks based on the infectious disease specialist’s recommendation. Surgical treatment with incision and drainage or aspiration is indicated if an abscess is detected in imaging, which will hasten recovery.

Anticoagulation in Lemierre’s disease remains controversial as large-scale randomized trials could not be performed due to the rarity of the disease. In most reported cases, low-molecular-weight heparin or warfarin was used for two weeks to six months. We started the patient on intravenous heparin followed by apixaban, which was given for a total of 12 weeks duration. Cupit-Link et al. [9] performed a retrospective analysis of 18 patients from January 1998 to December 2014. Out of the cohort, nine were female, nine were male, six were in the pediatric age range, and 12 were adults. All patients received broad-spectrum antibiotics; six received anticoagulation for four weeks, and 12 did not. Patients were followed for three months, and all have improved thrombi status. The authors have theorized that thrombosis should resolve if the infection is treated as the inciting event is an infection. Patients with progression of disease despite optimal antibiotic therapy, intracranial extension of thrombus, or associated thrombophilia have shown a benefit of anticoagulation therapy. Rebelo et al. [10] recommended anticoagulation if it is not contraindicated. Anticoagulation therapy can shorten the duration of antibiotics by dissolving the clot and exposing bacteria entrapped in the thrombus. Performing a prospective control trial is difficult due to the rarity of the disease. A multicenter study, however, might shed more light on the effect of antibiotics and anticoagulation and the treatment duration, given the disease’s current emergence.

## Conclusions

Lemierre’s syndrome should be kept in the differential diagnosis of pharyngitis. Appropriate antibiotics, anticoagulation, and surgical management should be considered promptly. From our experience, it is suggested that anticoagulation be initiated as soon as possible if Lemierre’s disease is associated with an intracranial thrombus that persists even with antibiotic treatment if anticoagulation is not contraindicated or if there is another associated thrombophilia. The duration of antibiotics and anticoagulation should be tailored based on the patient’s clinical presentation and expert opinion. It is crucial to remember that organisms such as *Streptococcus anginosus* should be covered with broad-spectrum antibiotics before getting the final culture report. Extensive randomized studies are needed to formulate a proper guideline.

## References

[1] Eilbert W, Singla N (2013). Lemierre's syndrome. Int J Emerg Med.

[2] Allen BW, Anjum F, Bentley TP (2023). Lemierre syndrome. http://www.ncbi.nlm.nih.gov/books/NBK499846/.

[3] Karkos PD, Asrani S, Karkos CD, Leong SC, Theochari EG, Alexopoulou TD, Assimakopoulos AD (2009). Lemierre's syndrome: a systematic review. Laryngoscope.

[4] Chirinos JA, Lichtstein DM, Garcia J, Tamariz LJ (2002). The evolution of Lemierre syndrome: report of 2 cases and review of the literature. Medicine (Baltimore).

[5] Osman M, Hasan S, Bachuwa G (2017). Oesophageal cancer presenting as Lemierre's syndrome caused by Streptococcus anginosus. BMJ Case Rep.

[6] Dalen CT, Mekhail AM (2015). Lemierre syndrome: early recognition and management. CMAJ.

[7] Riordan T, Wilson M (2004). Lemierre's syndrome: more than a historical curiosa. Postgrad Med J.

[8] Johannesen KM, Bodtger U (2016). Lemierre's syndrome: current perspectives on diagnosis and management. Infect Drug Resist.

[9] Cupit-Link MC, Nageswara Rao A, Warad DM, Rodriguez V (2017). Lemierre syndrome: a retrospective study of the role of anticoagulation and thrombosis outcomes. Acta Haematol.

[10] Rebelo J, Nayan S, Choong K, Fulford M, Chan A, Sommer DD (2016). To anticoagulate? Controversy in the management of thrombotic complications of head & neck infections. Int J Pediatr Otorhinolaryngol.

